# Blood concentrations of per- and polyfluoroalkyl substances are associated with autoimmune-like effects in American alligators from Wilmington, North Carolina

**DOI:** 10.3389/ftox.2022.1010185

**Published:** 2022-10-20

**Authors:** T. C. Guillette, Thomas W. Jackson, Matthew Guillette, James McCord, Scott M. Belcher

**Affiliations:** ^1^ Department of Biological Sciences, North Carolina State University, Raleigh, NC, United States; ^2^ Center for Environmental Measurement and Modeling, Office of Research and Development, U.S. Environmental Protection Agency, Durham, NC, United States

**Keywords:** autoantibodies, autoimmune, crocodilian, immune toxicity, lupus, one health, PFAS

## Abstract

Surface and groundwater of the Cape Fear River basin in central and coastal North Carolina is contaminated with high levels of per- and polyfluoroalkyl substances (PFAS). Elevated levels of PFAS have also been found in blood of fish and wildlife from the Cape Fear River, and in the blood of human populations reliant on contaminated well or surface water from the Cape Fear River basin as a source of drinking water. While the public and environmental health impacts of long-term PFAS exposures are poorly understood, elevated blood concentrations of some PFAS are linked with immunotoxicity and increased incidence of some chronic autoimmune diseases in human populations. The goal of this One Environmental Health study was to evaluate PFAS exposure and biomarkers related to immune health in populations of American alligators (*Alligator mississippiensis*), a protected and predictive sentinel species of adverse effects caused by persistent toxic pollutants. We found that serum PFAS concentrations in alligator populations from the Cape Fear River were increased compared to a reference population of alligators from the adjoining Lumber River basin. The elevated serum PFAS concentrations in the Cape Fear River alligators were associated with increased innate immune activities, and autoimmune-like phenotypes in this population. In addition to evidence of significantly higher double stranded-DNA binding autoantibodies in adult Cape Fear River alligators, our qRT-PCR analysis found remarkably high induction of Interferon-α signature genes implicated in the pathology of human autoimmune disease. We interpret the association of increased PFAS exposure with disrupted immune functions to suggest that PFAS broadly alters immune activities resulting in autoimmune-like pathology in American alligators. This work substantiates and extends evidence from experimental models and human epidemiology studies showing that some PFAS are immune toxicants.

## Introduction

Per- and polyfluoroalkyl substances (PFAS) are a class of synthetic organic chemicals that are global contaminants of both built and natural environments (OECD 2018). Owing to their extensively fluorinated aliphatic backbone, PFAS are chemically stable, resistant to thermal and enzymatic breakdown, and can persist in terrestrial and aquatic environments. Because of their widespread use over the past 70-plus years, many PFAS and their terminal breakdown products have become ubiquitous contaminants of the land, water, and air through which humans and wildlife are exposed (De Silva et al., 2021). In the US alone, drinking water supplies for an estimated 200 million people are contaminated with PFAS, with recent analyses estimating the direct annual health-related cost due to PFAS exposure ranges from $37–59 billion in the US and €52–84 billion per year in the European Union (Andrews and Naidenko, 2020; Cordner et al., 2021).

Currently, the US EPA has set reference doses (RfD) for human health toxicity for four PFAS including GenX chemicals (3 × 10^−6^ mg/kg-day), PFBS (3 × 10^−4^ mg/kg-day), PFOA and PFOS (2 × 10^−5^ mg/kg-day for both) (US EPA, 2021). Evidence from both experimental animal and human epidemiologic studies has linked increased exposure to the most abundant PFAS with immunotoxicity and altered immune functions (DeWitt, 2015). Those data led the United States National Toxicology Program to conclude that perfluorooctanoic acid (PFOA) and perfluorooctanesulfonic acid (PFOS) are hazards to the human immune system (DeWitt, 2015; NTP, 2016). Additional epidemiologic findings have also demonstrated associations of PFAS exposures with immunosuppression and adverse health impacts that include an increased incidence in childhood infection, decreased antibody production in response to vaccination, and increases in severity of COVID-19 (Grandjean et al., 2020; Fenton et al., 2021). There is also evidence from human studies that links some PFAS exposures with an increased incidence of chronic autoimmune disorders, including thyroid disease and inflammatory bowel diseases (Fenton et al., 2021). The mechanisms of PFAS-mediated immunotoxicity and their roles in autoimmunity are poorly understood.

**FIGURE 1:**
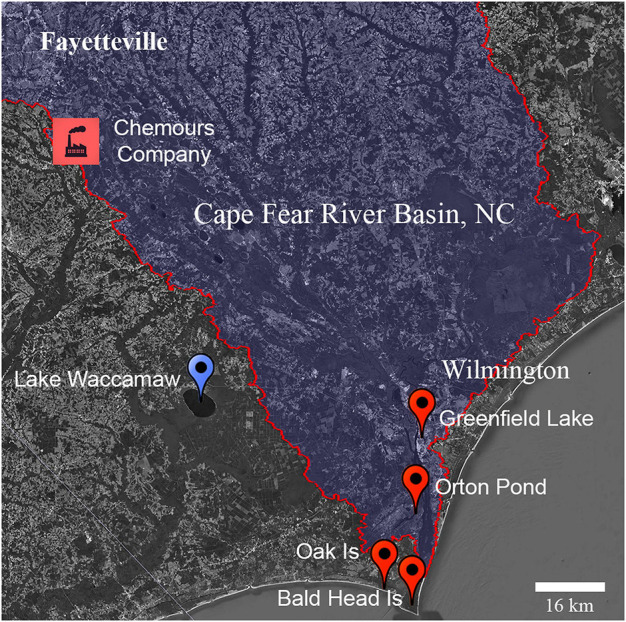
Location of study sites in South Eastern North Carolina. Location of alligator sampling sites at Lake Waccamaw (blue) and from the Cape Fear River (orange). The location of known upstream fluorochemical production discharge is indicated.

The Cape Fear River basin, located in central and coastal North Carolina, encompasses over 9,300 sq. miles of waterways that service ∼5.2 million people in rural and urban communities, and is typical in regard to contaminant burden of many regions with high PFAS contamination of surface, ground, and drinking water (Figure 1). Upstream PFAS contamination in the Cape Fear originates primarily from fluorochemical production, manufacturing, wastewater treatment discharges, and the use of aqueous film forming foams (AFFF) as fire suppressants (McCord and Strynar, 2019). In 2017, surface water sampling from the Cape Fear River revealed high levels of per- and polyfluoroether acids (PFEAs) and fluoropolymer manufacturing byproducts with diverse chemical structures originating from a fluorotelomer and fluoropolymer PFAS production facility active since the early-1980s (Sun et al., 2016; Hopkins et al., 2018). Both novel perfluoroalkyl ether acids (PFEA) such as HFPO dimer acid (HFPO-DA or GenX), Nafion byproducts, and a variety of other fluorochemicals from PFAS-based AFFF used as a fire suppressant by military, municipalities, and regional airports have now been detected in the drinking water and blood samples of residents living near the Cape Fear River (McCord and Strynar, 2019; Kotlarz et al., 2020; Ruyle et al., 2021). Further, total organic fluorine analysis detected extremely high sum PFAS concentrations in excess of 100,000 ng/L in the Cape Fear River at drinking water treatment plant intakes, suggesting that humans and aquatic ecosystems in the Cape Fear River basin have been experiencing very high levels of total PFAS exposure (Zhang et al., 2019). The potential for adverse impacts on both ecosystems and human populations from this contamination has been confirmed by wildlife and human population based PFAS exposure studies that found increased levels of PFAS in fish, birds, and humans living near the Cape Fear River (Guillette et al., 2020; Kotlarz et al., 2020; Robuck et al., 2021). The public and environmental health impacts of long-term exposures resulting from poorly documented PFAS discharge into the lower Cape Fear are largely unknown.

The American alligator has been used for more than four decades as an aquatic biomonitoring and predictive sentinel species of adverse outcomes resulting from the integrated effects of persistent toxic chemicals (Crain and Guillette 1998; Guillette et al., 2000; Pérez and Wise 2018). Alligators living along the Cape Fear River are non-migratory apex predators with a life span exceeding 60 years that co-utilize human habitats contaminated with PFAS (Elsey et al., 2019). The aim of this study was to evaluate exposures and impacts of long-duration PFAS exposure on biomarkers of immune health in American alligators (*Alligator* mississippiensis), a top trophic carnivore at the human/wildlife interface of both rural and urban environments (Beal and Rosenblatt, 2020; Somaweera et al., 2020). Because the innate immune system is conserved across taxa and it is generally robust and highly protective in alligators (Merchant and Britton, 2006; Finger and Gogal, 2013), we predicted that alligators would serve as sensitive sentinels of cumulative adverse immune health resulting from PFAS exposures common to humans living in the Cape Fear River basin.

Building on previous biomonitoring studies demonstrating utility of American alligators as effective indicators of environmental PFAS contamination (Bangma, et al., 2017a; 2017b), the objective of our study was twofold: 1) characterize and compare the PFAS serum concentration profiles of populations of alligators living in the Cape Fear River basin and surrounding coastal waters and a site in the neighboring Lumber River basin, and 2) comparatively evaluate associations between PFAS concentrations and immune health endpoints in alligators from these two watersheds. Based on our previous research that indicated a positive association between serum PFOS concentrations and serum lysozyme, an important marker of the innate immune activity, in another aquatic predator, the Striped bass (Guillette el al, 2020), we expanded further the immune endpoints examined in this study to include biomarkers of humoral innate immunity (complement) and the adaptive immune system function, such as white blood cell counts and differentials, the presence of double-stranded DNA (dsDNA) autoantibodies, and *IFN-α* responsive gene expression.

## Materials and methods

### Animals and sampling procedures

All animal procedures were performed with approval by the North Carolina State University Institutional Animal Care and Use Committee (protocol #18-085-O). Alligators were sampled using active capture methods that employed snatch hooks from locations with direct access to the Cape Fear River and a site from the adjacent Lumber River basin, Lake Waccamaw, NC (34.044528, −77.9839; Figure 1). Lower Cape Fear River sites included: Greenfield Lake (34.21036, −77.93676) Wilmington, NC; Orton Pond in Brunswick County (34.11199, −77.95287); Oak Island, NC (33.90808, −78.06721), and Bald Head Island, NC (33.86451, −77.99453). Sampling occurred in 2018 (July 24 to October 25) and 2019 (April 18 to October 14).

Immediately following capture, a 10–15 ml whole blood sample was collected from the post-occipital spinal venous sinus using a sterile 20 or 18 g needle and a 30 ml syringe (Myburgh et al., 2014). Whole blood samples were transferred to 8 ml serum and lithium heparin coated plasma tubes (Vacutainer, BD, Franklin Lakes, NJ). Serum tubes were incubated for 30 min at ambient temperature to allow clot formation and then stored on ice. Plasma tubes were inverted gently following collection, then stored on ice until centrifugation. After field collections, blood was centrifuged (1800 x g for 10 min at 4°C) and serum/plasma fractions were immediately aliquoted into Teflon-free cryovials and stored at −80°C until analysis. Following the blood sample collections, alligators were visually examined for general health, and external injuries were noted and photographed. Sex was determined by cloacal examination with total length, snout to vent length (SVL), and tail girth measurements recorded. Following sample/data collection, alligators were released at the site of capture. Typical time from first contact to release of animal was dependent on animal size with a usual time to release being <15 min. The average time until completion of blood sampling was 7.1 min (SD = 7.8, *n* = 43).

### Chemicals and reagents

Methanol (Optima®, Lot 183,859), ammonium formate (99%, AC401152500), acetonitrile (ACN; Optima®, Lot 184819), and formic acid (99.5%, A117-50) from Fisher Scientific (Waltham, MA, United States) was used for extractions and instrument solvent gradient. All water used for extraction, in aqueous buffers and solutions were prepared in sterile Milli-Q A10 water (18Ω; 3 ppb total oxidizable organics), with the purified water analyzed for PFAS contamination prior to use. All laboratory glassware was rinsed with sterile Milli-Q water and methanol prior to use. The control material for the instrument methods was National Institute of Standards and Technology (NIST) Standard Reference Material (SRM) 1957 organic contaminants in non-fortified human serum. Chemicals for buffers and other standard laboratory chemicals were also purchased from Fisher Scientific and as indicated below.

### LC-MS analysis of serum PFAS concentrations

Matrix matched (serum) calibration solutions (*n* = 23; ranging from 0.1 ng/ml to 100 ng/ml) were prepared from neat standards in charcoal stripped fetal bovine serum (Life Technologies, Grand Island, NY; cat ^#^10437, Lot ^#^1754113; total protein 3.7 g/dl). An internal standard (IS) solution was prepared by diluting a solution containing 10 mass-labeled PFAS in water with 0.1 M formic acid (99.5%, A117-50, Fisher Scientific, Waltham, MA): ^18^O_2_-PFBS,^13^C_4_-PFBA, ^18^O_2_-PFHxS, ^13^C_4_-PFOA, ^13^C_4_-PFOS, ^13^C_5_-PFNA, ^13^C_9_-PFDA, ^13^C_4_-PFBA, ^13^C_2_-PFHxA, ^13^C_2_-6:2FTS (Wellington Labs, Guelph, ON). An additional matrix matched quality control (QC) sample was made by combining randomly selected aliquots of sampled American alligator serum (*n* = 12). The resulting alligator QC sample and NIST SRM 1957 (*n* = 6) was used to test the method for reproducibility and stability (Supplementary Table S2). Charcoal stripped fetal bovine serum was also spiked with 5 ng/ml of PFAS standards and used as an internal control for accurate measurement (*n* = 4). Additional control samples included fetal bovine serum method blanks (*n* = 7), and purified water field blanks (*n* = 5) made by injecting 4 ml of Milli-Q water into vacutainer tubes during field sampling on 5 different days during the sampling period.

All control and experimental samples were extracted and analyzed using methods identical to those detailed in our previous studies (Guillette et al., 2020). Briefly, 50 μl of each sample was aliquoted into a 2 ml polypropylene tube to which 100 μl of 0.1 M formic acid and internal standards (12.5 ng) were added. Ice-cold ACN (450 μl) was added to the tube and then vortexed for 3 s. Samples were then centrifuged at 12,500 x g for 5 min at room temperature. The resulting supernatant (100 μl) was collected and added to 300 μl of aqueous 0.4 mmol ammonium formate and then transferred to polypropylene autosampler vials. PFAS were analyzed using a Vanquish UPLC system (Thermo Fisher Scientific, Waltham, MA, United States) equipped with an Accucore C18 + column (2.1 mm × 100 mm x 1.5 μ) at a flow rate of 300 μl/min, injection volumes of 100 μl, and a binary mobile phase gradient composed of 95:5 H_2_O:ACN, 0.4 mM ammonium acetate and 95:5 ACN:H_2_O, 0.4 mM ammonium acetate. A Thermo Orbitrap Fusion mass spectrometer (Thermo Fisher Scientific, Waltham, MA) with a heated electrospray ionization (HESI) source operated in negative mode was used to detect PFAS. For compound validation, data was collected in data dependent mode with a preferred ion list consisting of the quantitated PFAS standards (Supplementary Table S1). PFAS quantitation was based on an eight-point calibration curve with three injections per concentration randomly assigned in the sample run of the internal standard normalized integrated peak area of the extracted ion chromatogram of the (M-H)-ion with a 5-ppm mass tolerance. The *r* ^2^ of all calibration curves used for analysis were above 0.97; limits of detection (LOD) were defined as the estimated mean concentration of method blanks plus three standard deviations (Supplementary Table S2). Multiple replicates of SRM 1957 (*n* = 6) were compared to the values on the Certificate of Analysis for the NIST SRM 1957 standards and were within 15.89% of expected values (Supplementary Table S4).

### Lysozyme assay

The EnzChek® Lysozyme Assay (Thermo Fisher Scientific, Waltham, MA, Cat. E-22013) was used according to manufacturer’s protocols to determine lysozyme activity in serum of a subset of American alligator serum samples (Lake Waccamaw: *n* = 22; 17 males and 5 females; SVL *M* = 98.0 cm, *SD* = 23.8; Cape Fear River: *n* = 24; 12 each sex; SVL *M* = 96.6, *SD* = 25.0). Samples were diluted 1:5 in sample buffer and analyzed in triplicate, the average relative standard deviation (RSD) for QC and experimental samples were 7% and 9%, respectively.

### Alligator serum complement assay

Complement activity was determined using a modified sheep red blood cell lysis assay previously used in alligators to assess relative complement activity (Merchant and Britton 2006). Packed Sheep red blood cells (RBCs, Innovative Research Novi, MI) were aliquoted into 15 ml polypropylene conical tubes and centrifuged for 5 min at 800 x g. Cell pellets were washed with phosphate buffered saline (pH 7.4; PBS) and then gently suspend in PBS and recentrifuged. Washed cells were resuspended at a final concentration of 2% RBCs in PBS or PBS supplemented with a final concentration of 50 mM EDTA. Cells were rocked gently and 150 μl of the cell suspension was rapidly aliquoted into 1.7 ml microcentrifuge tubes containing 150 μl of each alligator serum sample and mixed by inversion. Control experiments with plasma samples were analyzed in parallel and yielded comparable results. Because sufficient volumes of plasma were not available for many sampled animals, serum was used for the analysis. All sample aliquoting for each analysis was completed in ≤2 min. Mixed samples were incubated at room temperature for 20 min and then rapidly placed onto ice/water bath for 10 min, samples were centrifuged at 3,000 x g for 5 min and then rapidly returned to ice. Supernatant (100 μl) was aliquoted in duplicate into clear 96-well microtiter plates. Absorbance at 540 nm was determined using a Tecan microplate reader (Tecan Systems Inc., San Jose, CA, United States). Controls on each analysis plate included a blank control containing 1x PBS only, 1% RBC in PBS, 1% RBC in 1x PBS supplemented with 50 mM EDTA, and a positive control of maximum RBC lysis containing 2% RBCs to which an equal volume of deionized water was added. Percent maximal complement activity for each sample was calculated by subtracting the mean absorbance from the EDTA containing samples from the mean absorbance of the sample lacking EDTA. That difference was divided by mean maximal absorbance of the positive control samples, and the product was multiplied by 100. Each sample was analyzed a minimum of two times. The mean % CV ± SD for maximal RBC lysis positive controls was 1.75% ± 1.42% and for all experimental samples was 3.41% ± 4.43%.

### dsDNA ELISA analysis

Individual wells of 96 well microtiter plates were coated overnight at 4°C with 500 ng of double stranded calf thymus DNA suspended in DNA coating solution (Pierce, Rockford IL, cat # 17250). Plates were washed extensively with PBS containing 0.05% Tween 20 (Fisher Scientific), incubated at room temperature for 2 h in blocking buffer consisting of PBS supplemented with 10% bovine serum albumin (BSA; fraction V, Fisher Scientific), and then rewashed with PBS containing 0.05% Tween 20. Alligator plasma samples were diluted 1:500 and 1:1,000 into PBS containing 10% BSA. Diluted samples (100 μl) were added to wells in duplicate or triplicate and incubated overnight at 4°C. Wells were then washed extensively with PBS/0.05% Tween 20, blotted dry by inversion onto paper towels, and then incubated for 1 h at room temperature with affinity purified polyclonal horse radish peroxidase (HRP) conjugated goat anti-alligator IgG antiserum (Bethyl Labs, Montgomery, TX; Cat # A140-119P; Lot# A140-119P-6) that was diluted 1:10,000 (0.1 μg/ml) in PBS 10% BSA. Samples were extensively washed with PBS 0.05% Tween 20, blotted dry, and 50 μl of 1x TMB Solution (Invitrogen, Cat # 00-4201-56) was added to each well. The peroxidase reaction was terminated following incubation for 30 min at room temperature by the addition of an equal 50 μl volume of 2M sulfuric acid, and the absorbance of each well measured at 450 nm. Control samples on each analyzed plate included calf serum diluted 1:500 (Gibco), blocking buffer only, 1:500 dilution of alligator plasma or calf serum sample lacking HRP-conjugated anti-alligator antiserum, and coated wells lacking DNA. Following optimization of alligator plasma concentrations and alligator antiserum dilutions, specificity of DNA binding was confirmed by observing significant decreases in immunoreactivity in DNA-coated wells that were treated with DNase1 (10 units; RQ1 Dnase, Promega Madison, WI), and in samples of alligator plasma pretreated with 100 μg of calf thymus DNA. Experimental plasma samples were analyzed in duplicate or triplicate on a single plate at 1:500 dilution and on the same plate using an independent dilution of 1:1,000, with each analysis independently replicated. Representative samples with sufficient volume and quality of plasma from 4 females and 4 males from Cape Fear River and Lake Waccamaw were analyzed (Supplementary Table S2). The average intra-assay coefficient of variation ±SD for all experimental samples was 5.9% ± 1.4% (1:500) and 4.5% ± 1.1% (1:1,000). The average between plate coefficients of variation ±SD were 7.0% ± 1.7% (1:500) and 6.0 ± 1.5% (1:1,000). The average absorbance 450 nM ± SD observed for each sample diluted at 1:500 was 2.05 ± 0.02 times greater than samples diluted at 1:1,000.

### Whole blood cell counts and leukocyte differential

Blood smears were prepared from ∼10 μl of peripheral whole blood taken from a subset of alligators (Supplementary Table S2). Blood smears were prepared in the field and allowed to air dry at room temperature on Superfrost plus slides (Fisher Scientific, Pittsburgh, PA, United States). Slides were stained with Hemacolor® according to the manufacturer’s protocols (Sigma-Aldrich, St. Louis, MO, United States; Cat: 1.11661). Peripheral whole blood cell counts were made by evaluation of stained blood smears using 40X and 100X objectives on a Nikon Eclipse 80i microscope (Nikon; Melville, NY, United States). Each slide was examined independently by three different investigators blinded to sampling site, size, and sex. Leukocyte identity was determined by cellular morphology and staining characteristics (Stacy et al., 2011; Sykes and Klaphake 2015). A minimum of 400 leukocytes and thrombocytes were counted to calculate percentage of each cell type. Leukocytes were categorized into lymphocytes, basophils, heterophils, eosinophils, azurophils, and basophils with percent of each cell type determined by dividing the number of each cell type by the total cells counted minus the number of thrombocytes and then multiplying by 100. Variation between individual researcher counts was determined to be less than 7.9% for each cell type.

### TBARS assay

Plasma lipid peroxidation was analyzed for 100 μl alligator plasma samples ran in duplicate using TBARS (TCA method) Assay kit (#700,870, Cayman Chemical, Ann Arbor, MI) according to manufacturer’s supplied protocols. Malondiadehyde concentrations were calculated by interpolation from a standard curve generated by serial dilution of manufacturer supplied standards.

### Quantitative RT-PCR analysis

Total RNA from archived (stored at −80°C) American alligator whole blood was isolated using the Quick-RNA Whole Blood Kit (Zymo Research, Irvine, CA, United States). Samples from both Lake Waccamaw and Cape Fear River were selected randomly based only on the availability of samples. One µg of RNA was reverse transcribed using the high-capacity cDNA reverse transcription kit following manufacturer’s recommendations (Applied Biosystems; Grand Island, NY, United States). Standard Fast TaqMan PCR amplification was performed in triplicate on a Step One Plus Real-Time PCR System (Applied Biosystems; Grand Island, NY, United States) in a final volume of 20 µL containing ∼10 ng of cDNA (1.5 µl of RT product), 1x Universal Master Mix and custom TaqMan expression assay primers specific for each target alligator mRNA (Supplementary Tables S5, S6; Applied Biosystems; Grand Island, NY, United States). Relative expression was quantified using the 2^ΔΔCt^ method, in which ΔΔCt is the normalized value. Alligator *Gapdh* expression was used as an independent reference gene for normalization.

### Data and statistical analysis

Each animal/blood sample was assigned a randomized numeric code by NC State University researchers at the time of collection. Samples were decoded for site and length/size/sex only after PFAS measurement/analysis and biological markers analysis were completed. Blood slides were further deidentified to mask sample identity for whole blood cell counts. Samples used for each analysis were randomly selected based only on availability of sufficient material for analysis, and when possible to balance analysis for comparable numbers of males and females, life stage (adult SVL >90 cm; juvenile SVL <90 cm, and body mass index (BMI) estimated using the formula BMI=Tail Girth/(SVL x 2) (Lawson et al., 2020). To control for possible effects of seasonality samples were date matched. Data was analyzed and visualized using Prism (version 9.3, GraphPad La Jolla, CA, United States); R statistical programming environment version 3.5.2; SPSS (Version 27.0, IBM, Armonk, NY, United States); JMP Genomics 9 (SAS, Cary, NC, United States).

For PFAS concentration data, means and concentration ranges were determined from values above the LOD and interpolated ([PFAS] = LOD/ $\sqrt{2}$ ) values were used when PFAS congeners were detectable but below the LOD [LOD = mean_blanks_ + 3(SD_blanks_)], non-detects were replaced with a value of zero. A Shapiro-Wilk’s test was used to assess normality of data; for data requiring normalization a natural log transformation was used, [PFAS] data was log_10_ transformed, and percentage/ratiometric data were arcsine transformed. Non-parametric statistics were used for data not meeting analysis model assumptions following data transformation. Multivariate general linear modeling was used to examine PFAS concentrations with site and sex as fixed factors and SVL, month, and year as covariates. Linear regression and Pearson’s correlation coefficient or Spearman’s rank correlation coefficient were used to calculate relationships between total PFAS serum concentrations and serum biomarkers. A 2-way analysis of variance (ANOVA) was used to evaluate peripheral blood counts data (cell type, location), and ∑log_10_ [PFAS] (SVL, location) data and biomarker activities were initially evaluated at each site using multivariate ANOVA (biomarker activity, ∑ log_10_ [PFAS], and/or location). For correlation analysis, individual analyte concentrations were transformed using equation Y = log10(Y+1). A minimal level of statistical significance for differences in values among or between groups was considered *p* ≤ 0.05.

## Results

### Analysis of serum PFAS concentrations

We used liquid chromatography and high-resolution mass spectrometry to determine concentrations of 23 different PFAS present in serum samples of 75 adult and juvenile alligators from North Carolina. Alligators were sampled beginning in July 2018 and continued through October of 2019 at sites experiencing both point and non-point source PFAS exposures along the Cape Fear River (Cape Fear River; n = 49), and from Lake Waccamaw (Lake Waccamaw; *n* = 26), a site <50 km away and in the adjoining Lumber River watershed with no known fluorochemical production (Figure 1; Table 1).

**TABLE 1:** Characteristics of alligators analyzed.

Characteristic	Total [n = (%)]	Cape Fear River	Greenfield Lake	Lake Waccamaw
Adult/Juvenile	75 (100)	49 (100)	26 (100)	26 (100)
Adult	41 (55)	27 (55)	14 (54)	22 (54)
Juvenile	34 (45)	22 (45)	12 (46)	12 (46)
Sex				
Female	26 (35)	19 (39)	12 (46)	7 (27)
Male	49 (65)	30 (61)	14 (54)	19 (73)
	Total Median (min-max)	Cape Fear River Median (min-max)	Greenfield Lake Median (min-max)	Lake Waccamaw Median (min-max)
SVL (cm)	93.3 (49.0–175.8)	95.0 (49.0–175.8)	90.8 (52.0–153.0)	92.7 (59.0–150.5)
Tail Girth (cm)	42.0 (20.2–103.2)	40.4 (20.2–103.2)	42.1 (21.4–71.0)	42.0 (25.8–74.0)
BMI	0.22 (0.19–0.29)	0.22 (0.19–0.29)	0.22 (0.20–0.28)	0.22 (0.21–0.25)

BMI: body mass index = Tail Girth/(2*SVL); SVL: snout to vent length; Adult: SVL >90 cm.

From the targeted list of 23 PFAS analyzed (Supplementary Table S1), we identified fourteen different PFAS, including long and short chain perfluoroalkyl acids (PFAA), perfluoroether acids (PFEA) and the fluorotelomer 6:2 FTS in these alligator blood samples (Supplementary Tables S2, S3). As anticipated from previous wildlife exposure studies along the Cape Fear River, PFOS was the predominant PFAS detected in alligator serum (Guillette et al., 2020; Robuck et al., 2021). We detected PFOS in 100% of samples analyzed and it accounted for 79.7% and 75.8% of the total PFAS present in alligator serum samples from the Cape Fear River and Lake Waccamaw respectively. The median number of PFAS congeners detected in serum samples from Cape Fear River alligators was 10 (range 4–12), whereas a median of 5 (range 2–9) was detected in Lake Waccamaw samples (Supplementary Table S3). The relative composition of long and short chain PFAAs and PFEAs detected in blood of Cape Fear River American alligators were in general agreement with those found in the blood of adults and children exposed to PFAS from drinking water in Wilmington, NC (Kotlarz et al., 2020).

**FIGURE 2:**
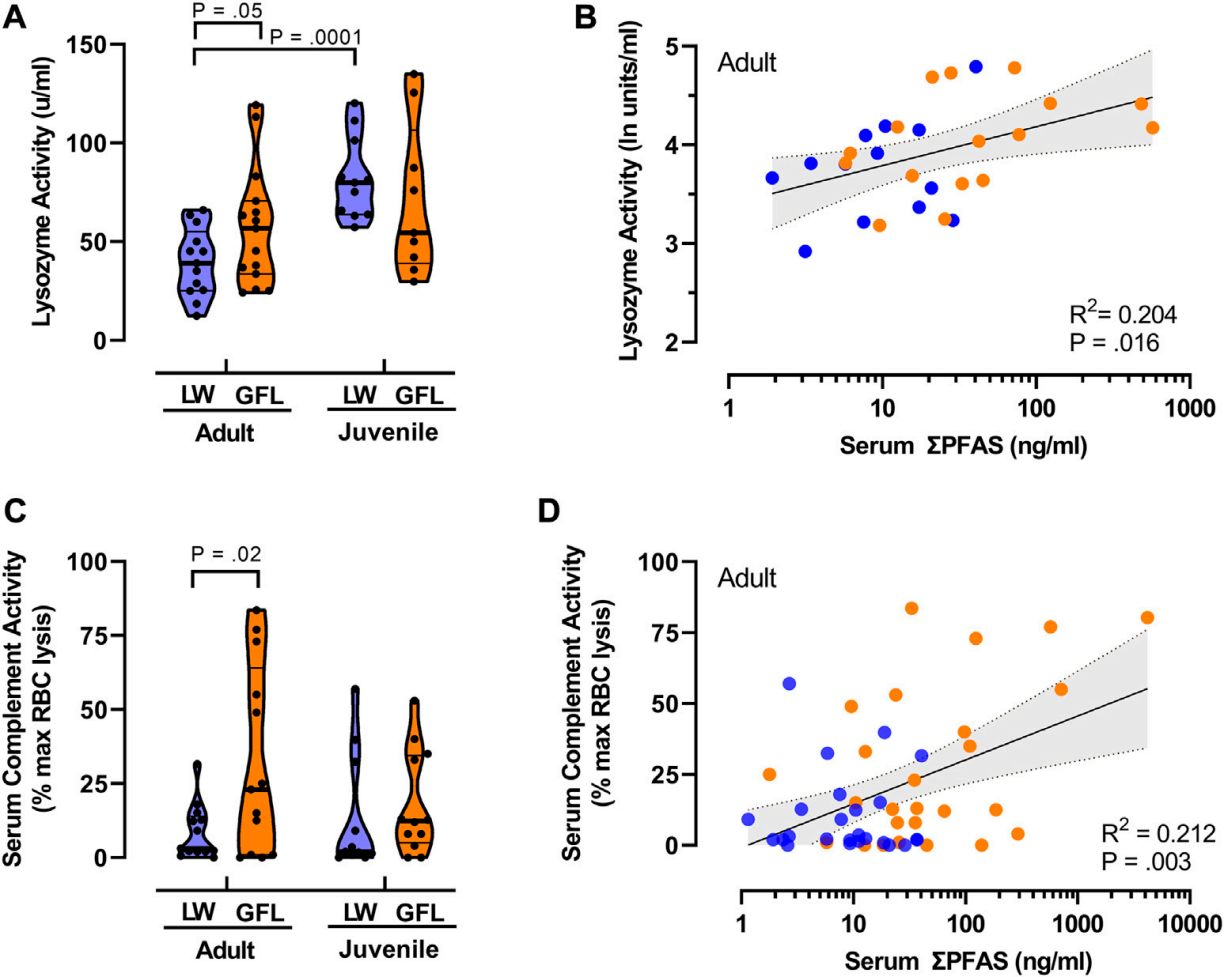
Relationships between PFAS exposure and innate immune functions. **(A)** Violin plots comparing median lysozyme activity in juvenile (SVL <90 cm) and adult American alligators (SVL >90 cm) from Lake Waccamaw (LW; thick lines indicate median; thin lines indicate quartiles) and from the Greenfield Lake (GFL) site on the Cape Fear River. Lake Waccamaw adult *n* = 13, juvenile *n* = 11; Greenfield Lake adult *n* = 15, juvenile *n* = 9. **(B)** Relationship between total PFAS concentrations and lysozyme activity in adult alligators. Solid line is the best fit linear regression, with shaded area represents 95% CI. *n* = 29. Reported *p*-value shown is for Pearson’s r. **(C)** Violin plots of complement activity in plasma samples of alligator from Lake Waccamaw (adult *n* = 13; juvenile *n* = 11) and Greenfield Lake (adult *n* = 13; juvenile *n* = 12). Shown is the percent of maximal EDTA sensitive sheep red blood cell lysis activity (thick lines indicate median; thin lines indicate quartiles). **(D)** The liner regression and correlation between total PFAS concentrations and complement activity in adult alligators. (*n* = 50). Reported *p*-value of Pearson’s r is shown.

Results of our general linear mixed effects modeling demonstrated a significant overall effect of location (F = 3.7, *p < 0.001*) on PFAS concentrations. No overall effects of sex or SVL on PFAS concentration were detected. There were no effects of sex on any PFAS, and the only effect of SVL was on HFPO-DA (F = 6.12, *p = 0.02*) and 6:2-FTS (F = 4.60, *p = 0.04*), both of which were undetected at Lake Waccamaw (Supplementary Table S7). There was an overall effect of the corrected model on PFBA (F = 4.27, *p < 0.001*), HFPO-DA (F = 3.52, *p < 0.001*), PFO4DA (F = 3.25, *p = 0.01*), PFHxS (F = 2.50, *p = 0.03*), Nafion bp-2 (F = 5.33, *p < 0.001*), PFO5DoDA (F = 2.77, *p = 0.02*), PFNA (F = 5.78, *p < 0.001*), PFDA (F = 3.53, *p < 0.001*), and PFOS (F = 40.46, *p < 0.001*). There was also an effect of location with significantly higher serum concentrations for samples collected from the Cape Fear River relative to Lake Waccamaw in PFBA (F = 6.99, *p = 0.01*), NVHOS (F = 4.52, *p = 0.04*), HFPO-DA (F = 4.54, *p = 0.04*), 6:2-FTS (F = 4.57, *p = 0.04*), PFHxS (F = 5.30, *p = 0.02*), PFOA (F = 5.30, *p = 0.02*), Nafion bp-2 (F = 23.2, *p = 0.04*), PFO5DoDA (F = 7.17, *p = 0.01*), PFNA (F = 23.72, *p < 0.001*), PFDA (F = 14.92, *p < 0.001*), and PFOS (F = 8.06, *p < 0.001*). These results are consistent with an exposure profile enriched for PFAS associated with chemical manufacturing facilities and other sources along the Cape Fear River (Sun et al., 2016; Pétré et al., 2021). Concentrations and detection frequencies of PFEAs (e.g. Nafion bp-2), PFAS congeners related to upstream production and discharge from the Chemours Fayetteville Works facility, were enriched in the Cape Fear River serum samples (Supplementary Table S2). By contrast the PFAS exposure profiles observed in blood of Lake Waccamaw alligators were characterized by the presence of bioaccumulative six-carbon and longer PFAAs (Figure 2B; Supplementary Table S8). However, the PFEAs PMPA (*n* = 4, 15%), PDO4DA (*n* = 6, 23%), and PFO5DoDa (*n* = 5, 19%) were detectable in some alligator serum samples from Lake Waccamaw, suggesting there is also low level PFEA contamination within the Lumber River basin ecosystem (Supplementary Table S2).

We next used correlation analysis to examine the hypothesis that PFAS exposures of alligators from the Cape Fear River were influenced by distance from upstream sources of PFAS contamination (Zhang et al., 2019; Pétré et al., 2021). Log_10_ transformed serum total PFAS concentrations and the distance downstream from a well-characterized sources of PFAS manufacturing (Fayetteville Works) were negatively correlated *r* (49) −0.34, *p* = 0.008. With the exception of PFDA, our correlation analysis found a moderate negative relationship between distance downstream and serum concentration of each detected PFAA (Table 2). The relationship between PFEA and distance downstream was weakly negative, but failed to reach significance, *r* (49) −0.229, *p* = 0.057, Nafion bp-2 was the only detected PFEA analyzed for which a significant correlation was identified, and a weakly positive relation was found for 6:2 FTS.

**TABLE 2:** Correlation of PFAS concentrations and distance from Fayetteville Works production facility.

Class	Spearman *r*	*p* value
∑ PFAS	**−0.343**	**0.008**
∑ PFEA	**−**0.229	0.057
∑ PFAA	**−0.465**	**0.0004**
Analyte(s)		
Class: PFAA		
PFBA	**−0.329**	**0.011**
PFHxA	**−0.317**	**0.013**
PFOA	**−0.439**	**0.0008**
PFNA	**−0.495**	**0.0002**
PFDA	0.012	0.478
Class: PFAS		
PFHxS	**−0.448**	**0.0006**
PFOS	**−0.459**	**0.0005**
Class: PFEA		
PMPA	0.231	0.056
NVHOS	**−**0.206	0.078
HFPO-DA	0.221	0.064
PFO4DA	**−**0.091	0.266
Nafion-bp2	**−0.296**	**0.019**
PFO5DoDa	0.070	0.318
Class: Fluorotelomer		
6:2 FTS	**0.260**	**0.036**

Analyte concentrations were transformed using equation Y = log10(Y+1); PFAA: perfluoroalkyl acids, PFEA, perfluoroalkyl ether acids; PFAS, perfluoroalkyl substances. Bold values indicate statistical significance.

**FIGURE 3:**
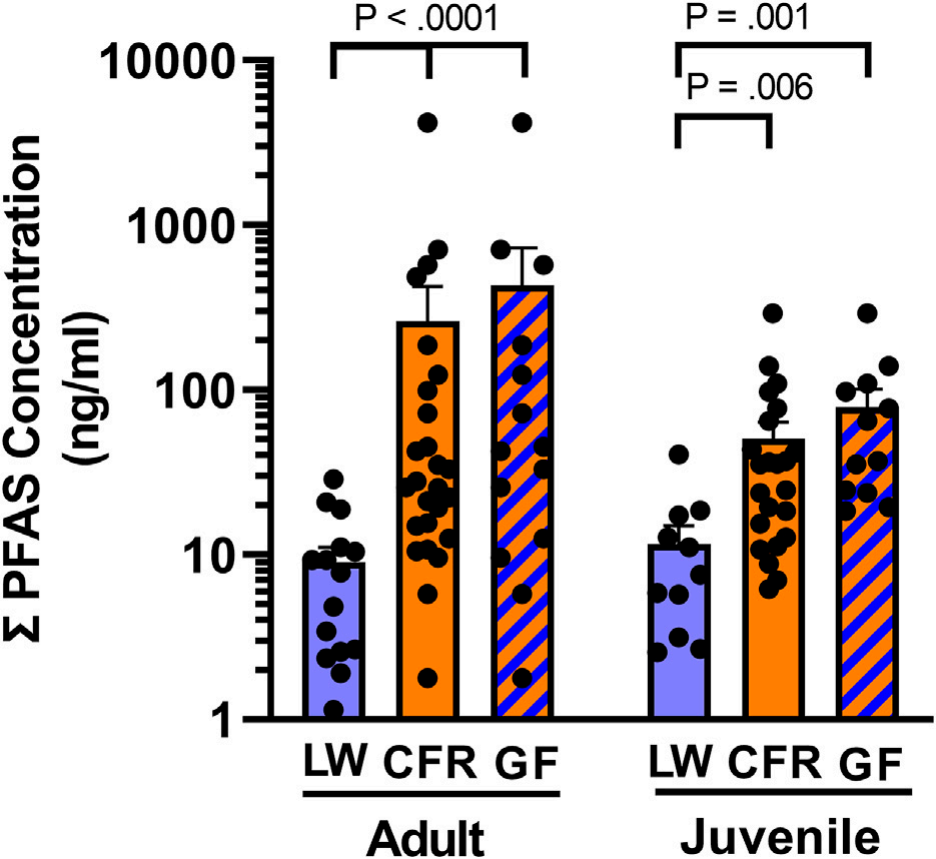
Total serum PFAS exposure. Mean of PFAS concentrations (ng/ml) found in serum of adult (SVL ≥90 cm) and juvenile American alligators from Lake Waccamaw (LW, adult n = 15; juvenile *n* = 11) and the Cape Fear River (CFR, adult n = 26; juvenile = 23), and the subset of animals from Greenfield Lake (GF, adult = 14; juvenile = 12).

As anticipated from the GLMM analysis, ANOVA found a significant effect of location on total serum PFAS concertation [F (1, 71) = 27.3, *p* < 0.0001], and did not detect differences in total PFAS concentrations between juveniles (SVL <90 cm) and adults [F (1, 71) = 0.0018, *p* = 0.965] at either site. There was not an interaction between groups [F (1, 71) = 0.989, *p* = 0.323]. Results of a protected Fisher’s LSD indicated that serum concentrations of PFAS were significantly greater in both the juveniles (*p* = 0.006) and adult alligators (*p* < 0.0001) from the Cape Fear River (Figure 3). Those findings were unchanged when we compared the ∑PFAS concentrations from the most upstream site at Greenfield Lake [F (1, 48) = 31.1, *p* < 0.0001]. There were no differences between size class [F (1, 48) = 0.08, *p* < 0.78] and the findings were not qualified by an interaction of site and size class [F (1, 48) = 0.26, *p* < 0.61].

### Associations between PFAS concentrations and innate immune activity

To avoid confounds related to decreasing PFAS concentrations at downstream sites on the Cape Fear River (Table 2), and differences in habitats, our analysis of lysozyme and complement activity as biomarkers of innate immune function focused on samples collected from Greenfield Lake (Figure 2). The results of our two-way analysis of variance comparing lysozyme activity with sampling site (Greenfield Lake, Lake Waccamaw) and size class (adult, juvenile) as between sample factors, found a main effect of size class [F (1, 44) = 13.35, *p* = 0.0007], the effect of sampling site was not significant [F (1, 44) = 0.14, *p* = 0.713]. However this was qualified by an interaction between sampling site and age class [F (1, 44) = 4.772, *p* = 0.03]. A Fisher’s LSD multiple comparisons test indicated that lysozyme activity of Lake Waccamaw juvenile alligators (*M* = 82.0 u/ml, SD = 20.7) was not significantly different than activity of Cape Fear River juveniles (*M* = 70.7 u/ml, SD = 38.5; *p* = 0.24). However activity in Lake Waccamaw juveniles was significantly greater than activity detected for adult alligators from both Lake Waccamaw (*M* = 39.6 u/ml, SD = 17.3; *p* = 0.001) and Greenfield Lake (*M* = 57.4 u/ml, SD = 29.9; *p* = 0.02). Lysozyme activity of Greenfield Lake adults was significantly higher than activity in adults from Lake Waccamaw (Figure 2A; *p* = 0.05). Those findings were similar to our previous findings that linked higher concentrations of PFAS with elevated lysozyme activity in striped bass (*Morone saxatilis*) from the Cape Fear River (Guillette et al., 2020). Using Pearson’s ranked correlation to assess the relationship between log_10_ transformed total PFAS concentrations and ln transformed lysozyme activity of adult alligators from both sites, we observed a positive correlation between serum PFAS and lysozyme activity, *r* (29) = 0.44, *p* = 0.016 (Figure 2B). We did not observe a similar correlation between PFAS concentrations and lysozyme activity in juveniles, *r* (19) = -0.24, *p* = 0.334).

Using two-way ANOVA of complement activity (Figure 2C), with sampling site (Greenfield Lake, Lake Waccamaw) and size class (adult, juvenile) as between sample factors, we also found a main effect of sampling site [F (1, 45) = 4.73, *p* = 0.035], the effect of size class was not significant (F (1, 45) = 0.42, *p* = 0.515), no interaction was observed [F (1, 45) = 1.022, *p* = 0.318]. Fisher’s LSD multiple comparisons test indicated that complement activity of Lake Waccamaw juvenile alligators (*M* = 13.5%, SD = 20.0) was not significantly different than activity of Greenfield Lake juveniles (*M* = 18.3%, SD = 14.5; *p* = 0.430). Complement activity was significantly greater in adults from Greenfield Lake (*M* = 31.9%, SD = 31.6; *p* = 0.025; Lake Waccamaw: *M* = 8.6%, SD = 9.2) (Figure 2C). We also found a positive correlation between serum PFSA concentrations and complement activity of adult alligators from both sites, Pearson’s *r* (29) = 0.44, *p* = 0.016 (Figure 2D).

### Phenotypes observed in alligators from the Cape Fear River

**FIGURE 4:**
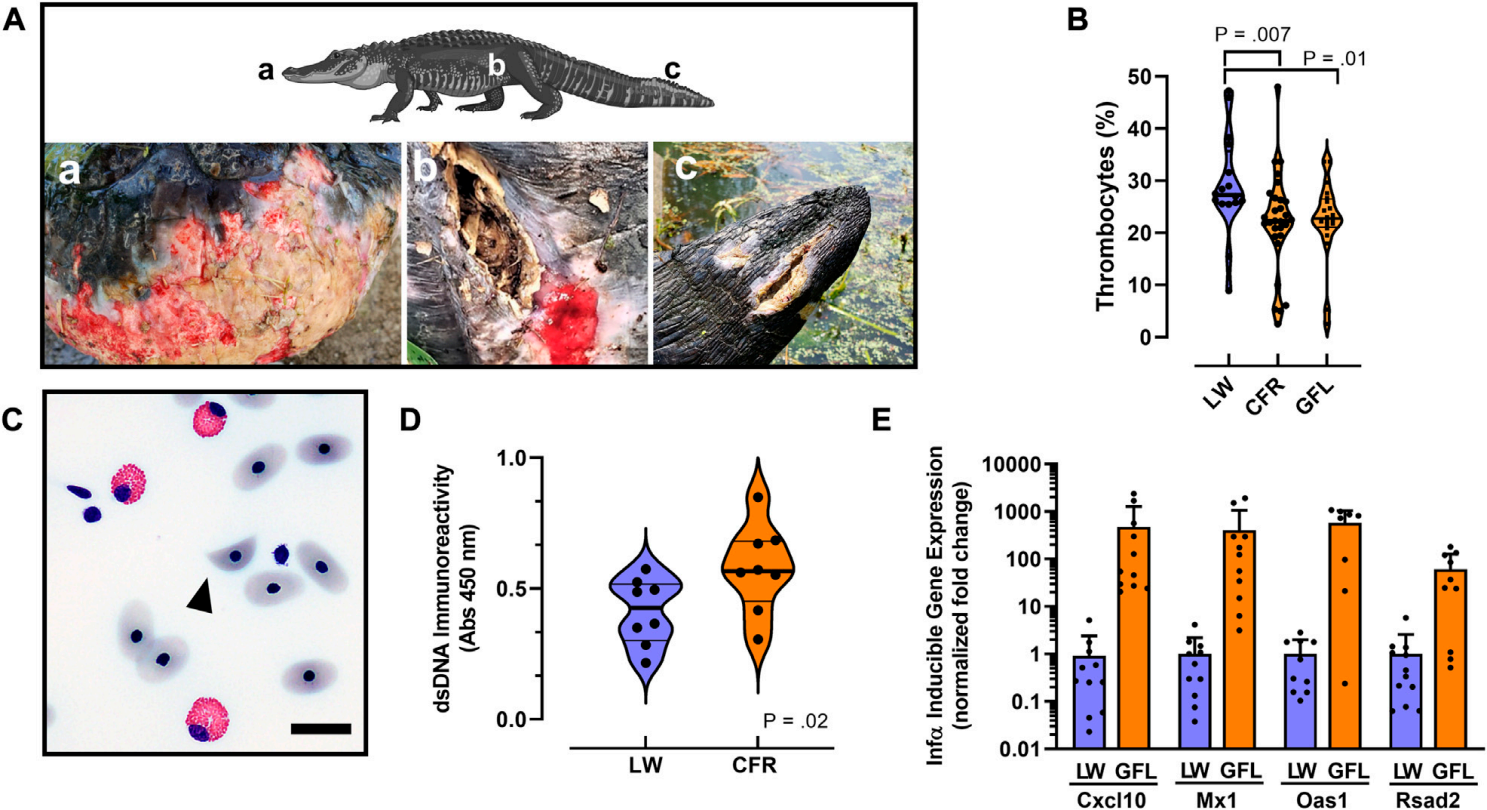
Autoimmune-like phenotypes associated with PFAS exposure. **(A)** Examples of cutaneous lesions and unhealed wounds associated with PFAS exposure in 3 representative animals sampled from the Greenfield Lake in 2019. Lower case letters indicate body location of lesion shown in each panel. **(B)** Violin plots comparing median thrombocyte numbers quantified from whole blood smears in alligators from Lake Waccamaw (*n* = 16), Cape Fear River (*n* = 39) and the subset from Greenfield Lake (*n* = 20). **(C)** Representative photomicrograph of a schistocyte (arrowhead) observed in a whole blood sample isolated from an alligator from Greenfield Lake. Bar = 10 μm **(D)** Violin plots comparing median anti-dsDNA immunoreactivity in male (*n* = 4) and female (*n* = 4) alligator serum samples from both sites. **(E)** Quantitative RT-PCR analysis of *IFN-α* signature gene expression analysis. Note the *X*-axis scale is log10 relative fold-change. All differences in expression are significant (*p* < 0.0001). For violin plots median is indicted by a thick line and thin lines indicate quartiles.

Despite living in environments with near-constant exposure to pathogenic microorganisms, and having especially high body burdens of fecal coliform and other pathogenic bacteria, crocodilians, including wild American alligators, rarely suffer from systemic or skin infections from these microorganisms (Johnston et al., 2010; Zimmerman et al., 2010; Keenan and Elsey, 2015). However, during our sampling we observed a notably uncharacteristic increase in incidence of skin lesions, unhealed and infected wounds in 7 of the 49 sampled Cape Fear River alligators (Figure 4A, Supplementary Table S2). Field observations of these lesions found purulent exudate and notable odor consistent with infection, and extensive slough (white/gray devitalized tissue and debris) was observed (Figure 4A). The presence and appearance of these lesions were reminiscent of slowed wound healing and vasculitis that is associated with some autoimmune diseases in humans (Shanmugam et al., 2017). Although fresh and healed wounds, including loss of limbs that were likely related to territorial and mating interactions, were observed in alligators sampled in both populations, similarly infected and unhealed wounds were not observed in alligators from the Lake Waccamaw site.

We also noted qualitative decreased clotting in some Cape Fear River alligators following blood collection (field observations); that phenotype was consistent with our findings of modestly decreased numbers of thrombocytes in whole blood cell counts from Cape Fear River alligators or only the population from Greenfield Lake (Figure 4B). A two-tailed Mann-Whitney test indicated that that numbers of thrombocyte present in blood samples from Lake Waccamaw were greater (*Mdn* = 27.25) than in samples from the Cape Fear River (*Mdn* = 22.30), *U* = 120.5, *p* = 0.007. Considering only samples collected from Greenfield Lake did not change the outcome of this analysis (Greenfield Lake: *Mdn* = 22.75, U = 120.5, *p* = 0.01).

### Serum lipid peroxidation and blood cell phenotypes

We compared the relative levels of lipid peroxidation products in plasma using a thiobarbituric acid reactive substance assay and found no evidence for increases in general systemic mechanisms of cellular injury and oxidative stress in the Cape Fear River alligators or the subset of alligators with highest PFAS concentrations from Greenfield Lake (Supplementary Figure S1; Lake Waccamaw *Mdn* = 1.60 μM, Cape Fear River *Mdn* = 1.51 μM; *U* = 535, *p* = 0.952; Greenfield Lake *Mdn* = 1.34 μM; *U* = 305, *p* = 0.421). Our analysis of differential white blood cell counts also found no evidence for a general increase in chronic stress; a Mann-Whitney test found no significant differences in percent of heterophiles in blood samples from Greenfield Lake (*Mdn =* 16.6%) and Lake Waccamaw (*Mdn* = 15.7%), *U* = 159, *p* = 0.09, and the heterophile/lymphocyte ratios from Greenfield Lake (*Mdn* = 0.336) and Lake Waccamaw (*Mdn* = 0.358) were not significantly different, *U* = 194, *p* = 0.378.

However, during our differential analysis of white blood cells, we observed a greater number of samples with abnormal red blood cell phenotypes including schistocytes (helmet cells) in samples from Cape Fear River alligators. These helmet cells were found in 65.5% of Cape Fear River samples analyzed (*n* = 29, Figure 4C), whereas none were found in the samples from Lake Waccamaw (*n* = 11; Supplementary Table S2). A Fisher’s exact test indicated that the observed number of samples from Cape Fear River alligators with schistocytes was significantly greater (*p* = 0.0002) compared to the samples from Lake Waccamaw.

### dsDNA binding antibodies and *IFN-α*-responsive autoimmune signature gene expression

Based on the elevated innate immune functions associated with elevated PFAS exposure, and the increased presence of unhealed wounds, helmet cells, and modestly lower numbers of thrombocytes of the Cape Fear River alligator population we next evaluated the hypothesis that hallmark phenotypes associated with human autoimmune disorders would be more prominent in the Cape Fear River alligators (Crow et al., 2019; Sirobhushanam et al., 2021). Using an alligator specific anti-double stranded DNA (dsDNA) immunoglobulin (IgG) binding enzyme-linked immunosorbent assay, we evaluated a subset of male and female alligators from the Greenfield Lake (*n* = 6) and a nearby site in Wilmington (*n* = 2), compared to samples from Lake Waccamaw (*n* = 8; Figure 4D; Supplementary Table S2). Our results found that dsDNA binding IgGs were significantly greater in Greenfield Lake alligator plasma samples (*M* = 0.577, *SD* = 0.167), *t* (14) = 2.22, *p* = 0.04, compared to Lake Waccamaw samples (*M* = 0.413, *SD* = 0.127).

Finally, our RT-PCR analysis of type 1 interferon (*IFN-α*) responsive gene expression in alligator whole blood samples found extremely high expression of four different *IFN-α* signature genes, which in humans are implicated in pathology of human autoimmune diseases including systemic lupus erythematosus (Crow et al., 2019). Relative to samples from Lake Waccamaw, *IFN-α* responsive gene expression was induced >60-fold for *Rsad2* (viperin), and >400-fold for *Cxcl10*, *Mx1*, and *Oas1* in Greenfield Lake alligator blood samples (Figure 4E; Supplementary Table S5). The finding of extremely high induction of lupus-signature gene expression in the Cape Fear River alligators supports a central role for excessive production of type I interferons in increasing the innate immune functions associated with elevated PFAS exposures. As a whole, we interpret the greatly increased *IFN-α* signature gene expression, elevated autoantibodies, increased numbers of skin lesions and unhealed wounds, and elevated innate immune activity observed in the Greenfield Lake population to suggest an elevated autoimmune-like activities, which might be related to increased PFAS exposure, in this population of American alligators.

## Discussion

Our work found increased ΣPFAS concentrations in American alligators sampled from the Cape Fear River. The median ΣPFAS concentrations found in alligator serum from the Cape Fear River (27.9 ng/ml; Supplementary Table S3 was >220 time higher than median ΣPFAS concentrations found in Cape Fear River water analyzed between 11 September 2018 and 18 November 2019 (*Mdn* = 126.1 ng/L; min = 39.95, max = 377.01; *n* = 78) at the Sweeney water treatment plant in Wilmington, NC (CFPUA, 2022). Whereas, surface water testing was not done for the “reference” site at Lake Waccamaw, located in the Lumber River basin, alligators sampled from this area had lower but evident concentrations of PFAS. This speaks to the global contamination documented in numerous studies, as well as widespread PFOS bioaccumulation in vertebrate aquatic species (Evich et al., 2022). The low levels of PFEA compounds found in Lake Waccamaw also highlights the mobility of these compounds, as this site is not on the same watershed as the proposed source of this contamination. Likely, low levels of PFEAs have migrated through rainfall, however other forms of transport including migration in groundwater cannot be ruled out.

The relative composition of long and short chain PFAAs and PFEAs detected in blood of Cape Fear River American alligators were in general agreement with those found in the blood of adults and children exposed to PFAS from drinking water in Wilmington, NC (Kotlarz et al., 2020). The concentrations of PFAS in serum sampled from Cape Fear River alligators is similar to levels found in the Wilmington human population (Kotlarz et al., 2020), but the median PFOS concentration was lower than those found in Striped bass (Guillette et al., 2020), seabirds caught off the coast (Robuck et al., 2021) and previous alligators caught at an AFFF-impacted sites (Bangma et al., 2017b).

Our qualitative field observations and quantitative laboratory assessment of phenotypes of altered immune activity were associated with increased PFAS concentrations in alligators sampled from Greenfield Lake, the site closest to the industrial sources of PFAS contamination. Like Striped bass caught within this ecosystem, PFAS concentrations in adult alligator serum were positively associated with lysozyme activity, a key component of anti-bacterial innate immune activity. Developmental toxicity studies in white leghorn chickens exposed *in ovo* to 0.9, 2.3, or 4.6 mg/kg PFOS egg weight, found increased lysozyme activity and other developmental impacts at all doses compared to control chickens (Peden-Adams et al., 2009). The serum average PFOS concentrations in the low dose group of chickens (153 ng/ml) were comparable to the average of serum PFOS concentrations found within the Cape Fear River alligators (133 ng/ml). In a 28 days acute toxicity study using B6C3F1 mice, significant increases in lysozyme were observed at an intermediate dose (0.1 mg/kg total administered dose) in females only, whereas lysozyme activity was increased in males at all doses analyzed, those increases failed to reach significance likely due to the small sample size (n = 5) analyzed (Peden-Adams et al., 2008). In wildlife studies, no significant age-adjusted associations between PFAS concentrations and lysozyme activity were found in adult Bottlenose dolphins (*Tursiops truncatus*) (Fair et al., 2013) and lysozyme was negatively associated with PFOS concentrations in juvenile loggerhead sea turtles (DeWitt, 2015). These findings suggest that impacts of PFOS exposure on lysozyme activity is dependent on dose, developmental timing and sex. Similarly, we did not observe a correlation between PFAS concentrations and lysozyme activity in juvenile alligators. Those differences could be related to other factors such as shorter duration of exposure, population density, or other factors including age or hormonal changes related to sexual maturity. Additional long-term biomonitoring, toxicokinetic, and immune-health studies are needed to elucidate the factors influencing the impacts of PFAS exposure on lysozyme activity, it is clear that changes in lysozyme activity alone are insufficient as a biomarker of PFAS-related immunotoxicity.

Field observations, suggestive of altered immune phenotypes, were documented in alligators sampled from Cape Fear River, these included increased incidence of atypical skin lesions, and unhealed and infected wounds in 7 of the 49 sampled Cape Fear River alligators (Figure 4A, Supplementary Table S2). Analysis of blood smears found evidence for site-specific differences in presence of a unique cell type not previously documented in any crocodilian species (Schistocyte). Schistocytes are fragmented red blood cells that in humans most often arise from mechanical shear forces caused by damaged endothelium and is a characteristic feature of microangiopathic hemolytic anemia (Zini et al., 2021). These abnormal blood cells are often associated with autoimmune disorders including thrombotic microangiopathy, and lupus nephritis in humans, but can also be found in blood smears of patients with other diseases (Tefferi and Elliott, 2004; Huh et al., 2013; Anders et al., 2020). Together those findings lead us to examine the hypothesis that serum PFAS concentrations were associated with autoimmune related impacts in these alligators.

Overall, our analysis found associations across multiple hallmark phenotypes of autoimmune related endpoints, some of which were associated with increased PFAS concentrations in American alligators from the Cape Fear River. The PFAS-associated changes in both the innate immune and adaptive immune system (increased presence of autoantibodies) may represent a significant health concern for wildlife and human health that may leave exposed populations at greater risk for infections and autoimmune disease. To the best of our knowledge, this is the first study documenting autoimmune-like activities in crocodilians, and evidence of an evolutionarily conserved mechanistic link between *IFN-α*-activation and expression of lupus-signature genes.

While both the environmental monitoring approach and health related findings are robust, there are potential limitations. In general, the number of samples is fairly small for a population based study and represent single biological measurements that only informs on concurrent exposure and health–related endpoints. As a result, the observed differences cannot be causatively linked to the noted differences in PFAS exposure. Whereas the untargeted analytic approach that we used for this study requires authentic standards for quantification of each reported analyte, it is not capable of identifying and quantifying all PFAS due to the large numbers of PFAS congeners known to be present in and around the Cape Fear River (Zhang et al., 2019; Kotlarz et al., 2020; Kirkwood et al., 2022). As a result, it is likely that we have underestimated PFAS exposure. Further, we have little understanding how other chemical contaminants and changing environmental and anthropogenic factors are impacting the immune health in these study populations. Additional sampling and analysis will be necessary to understand the full breadth of anthropogenic and environmental factors that are mediating the observed differences in immune-related endpoints.

Uniquely, for this work we implemented an integrated One Environmental Health approach using American alligators as sentinels to more fully understand the consequences of long-term exposure to complex and poorly understood mixtures of PFAS with the aim of improving health of impacted humans, animals, and ecosystems (Gibbs 2014; Pérez and Pierce Wise 2018). In addition to being long lived, and sharing their environment with humans, American alligators and other crocodilians, by virtue of having innate immune functions optimized to eliminate microbial pathogens, may serve as sensitive and predictive models for detecting autoimmune hazards caused by chemical pollutants. Our findings of potentially adverse immune-health effects in American alligators that have exposure profiles similar to the surrounding human population illuminates and reaffirms the need to reduce exposure and cease production and use of a chemical class that, through its ubiquity and persistence, is a global environmental health concern.

## Data Availability

The datasets presented in this study can be found in online repositories. The names of the repository/repositories and accession number(s) can be found in the article/Supplementary Material.
